# Crania Americana; or a Comparative View of the Skulls of Various Aboriginal Nations of North and South America

**Published:** 1840

**Authors:** 


### CRANIA AMERICANA; OR, A COMPARATIVE VIEW OF THE SKULLS OF VARIOUS ABORIGINAL NATIONS OF NORTH AND SOUTH AMERICA; TO WHICH IS PREFIXED AN ESSAY ON THE VARIETIES OF THE HUMAN SPECIES: illustrated by seventy-eight plates and a coloured map. By *Samuel George Morton*, M.D. Professor of Anatomy in Pennsylvania College, Philadelphia, &c. pp. 296, folio. J. Dobson, Philadelphia; Simpkin, Marshall and Co. London, 1839.

DR. MORTON'S method and illustrations in eliciting the elements of his magnificent Craniography, are admirably concise without being the less instructively comprehensive. His work constitutes, and will ever be highly appreciated in constituting an exquisite treasury of Facts well adapted, in all respects, to establish permanent organic principles in the natural history of Man.

While an extraordinary character is thus assigned to Dr. M.'s essay on the ground of its being a system of Facts, selected with discrimination and arranged with judgment, the inquiry may be made preliminarily—*What are Facts*, with a view to limit the term to a definite acceptation? Facts then are things accomplished so as to become deeds, or they are things discerned so as to be existences; and they present themselves to our attention under three distinct forms. 1. Facts are *Casual*, when their reality, as incidents, is admitted exclusively on the veracity of an observer and their own verisimilitude. 2. They are *Demonstrable* when, as circumstances or beings, they can be reproduced and ascertained by many different persons re-instituting the original processes of observation and experiment. 3. They are *Perceptible* when, as mental experiences, they can be renewed by many different persons repeating the original process of reflection on consciousness, by which they were first discovered. Now, in being manifest physical entities, the subjects of Dr. M.'s "comparative view" exhibit the genuine attributes of Facts, in being deposited in his own and other specified Collections accessible to the scrutiny of naturalists and physiologists retracing or extending the same course of investigation, they possess all the attributes of demonstrable Facts; and hence they will remain a splendid and imperishable monument of Dr. Morton's exertions for the advancement of Science, and an excellent authority to guide the researches of philosophers in determining the features and disposition of nations.

At the opening of a brief preface we find Dr. Morton stating that his chief design has been to give accurate delineations of the Crania of more than forty Indian nations, Peruvian, Brazilian and Mexican, together with an extended series from North America, from the Pacific Ocean to the Atlantic, and from Florida to the region of the Polar tribes. He has devoted especial attention to the singular distortions of the skull, caused by mechanical contrivances in use among various nations—Peruvians, Charibs, Natches, and the septs inhabiting the Oregon territory. His materials in this department are ample, and he represents them as having enabled him to give a full exposition of a subject which was long involved in doubt and controversy. Subsequently, he describes the processes by which these denaturalizing disfigurements are produced. Here however it may be cursorily noted, that these contrivances do not change the structure or qualities of the brain, but merely alter its form so as to give the head an unnatural shape, rendering it altogether an unfaithful or delusive source of observation for the Physiologist in conducting his researches, to establish characters of organic and mental distinction, in persons, families, and nations. In an introductory essay on the varieties of the Human species, Dr. Morton embodies a train of interesting practical observations. It is a remark of his, and he illustrates it—that the condition of man, under his infinitely varied circumstances, is less the effect of coercion than of choice; and, as we judge, the induction is confirmed by historical facts drawn from the traditions or records of every age and country. The doctor declines offering any solution of the motives which impel the native of a cold or hot, a temperate or torrid region, to prefer the dwellings or resorts of his fathers to all other lands, however pregnant with the seeds of happiness and comfort. In our matured opinion, this "choice" results entirely from the secret, and seldom perceptible, promptings of a primitive faculty of the mind, the innate love of home and country which intuitively cherishes the spirit of pure genuine patriotism.

Extensive emigrations, as Dr. M. thinks, have been mostly confined to the temperate zones, and to the civilized communities of modern times, in which the spirit of migratory enterprize is without limits. He adopts the opinion that, from remote ages, the inhabitants of every extended locality have been marked by certain physical and moral peculiarities, common among themselves and serving to distinguish them from all other people. Thus, at this time, the Arabians are precisely what they were in the days of the patriarchs; the Hindoos are altered in nothing since they were first described by the earliest writers: nor has any difference been made in the skin and hair of the Negro, by the influences of three thousand years: in like manner, the characteristic features of the Jews may be recognised in the sculpture of the temples of Luxor and Karnak, where they have been depicted for nearly thirty centuries. This identity of physical characteristics, preserved through numberless generations, and often under very dissimilar conditions, has occasioned various speculations respecting the origin of the human family. The prevalent belief is derived from the Sacred Writings; and, in their literal and obvious interpretation, these teach us that all men have descended from a single original pair. Hence, says Dr. M. it has been hastily and unnecessarily inferred, that the differences now observable in mankind are owing solely to vicissitudes of climate, locality, habits of life, and various collateral circumstances. Without attempting to pursue this intricate question in detail, he inquires whether it is not more consistent with the known government of the universe, to conclude that the same Omnipotence that created man would adapt him at once to the physical as well as the moral circumstances in which he was to dwell upon the earth? It is indeed difficult, he supposes, to imagine that an all-wise Providence, after having by the deluge destroyed all mankind excepting the family of Noah, should leave these to combat, and with seemingly uncertain means, the various external causes that tended to oppose the great object of their dispersion; and we are left to the reasonable conclusion, that each race was adapted from the beginning to its peculiar local destination: in other words,

Dr. M. assumes, that the physical characteristics which distinguish the different Races, are independent of external causes. Such appear in his mind, to have been the primitive distinctions among men ; but hostile invasions, the migratory habits of some tribes, and the casual dispersion into remote localities, have a constant tendency to confound these peculiarities ; and the proximity of two races has uniformly given rise to an intermediate variety partaking of the characters of both, without being identical with either : these are called *Mixed* races. Such is Dr. Morton's view of the rise of Races—a theme which has engaged the ingenuity of many naturalists, and proved the occasion of that diversity of opinion which is so frequent in human researches.

Instructed by authentic history in the Sacred Scriptures; we entertain a strong inclination to derive all the existing Races of Mankind from the three sons of Noah, "of whom was the whole earth overspread," and through whose " families, after their generations, the nations were divided upon the face of the earth, after the flood." With this exact authority then, we lean to the belief that the primary races of men were three—the Shemites, Hamites and Japhethites ; that, during the century intervening between the Deluge and the Dispersion at Babel, these original races would progressively divide into generic families, notwithstanding " the whole earth was of one language and of one speech ;" and that, after the Deity had " confounded their language that they could not understand one another's speech," their division into new tribes and families and races would be more frequent and numerous. We may also imagine that a supernatural distinction by physical and moral characters, giving origin to new races, would accompany the miraculous confusion of tongues—for, it is manifest, that such a result could easily be produced by the Divine Energy which created man, and endowed him with the wonderful attributes of body, life and mind, at the beginning. Another and, in our judgment, a principal efficient cause sufficiently well adapted to promote the multiplication of races, in primeval times, was the intermarriages of brothers with sisters through several generations, and thus impressing on their progeny the distinctive features of the Pair with whose segregation from their kindred this practice necessarily commenced. Illustrations of this process are unfortunately too evident in the facts of organic and mental peculiarities, often those of degeneracy, being propagated extensively by hereditary transmission. Thus far therefore, we agree with Dr. Morton in representing the physical characteristics which distinguish different races, as being mainly independent of external causes ; but, from the force of observation, experience, and history, we conclude that the agency of external causes does exert a decided, though imperceptible progressive influence in altering the human structure and economy.

Much erudition and research have been employed by physiologists, with a view to establish the *unity* of the Human species : the affirmative opinion, as Dr. M. observes, is maintained by Linnæus, Blumenbach, Cuvier, and many other distinguished naturalists : yet on the contrary, Virey has divided mankind into *two* species ; Dumoulins, into *eleven* ; and Bory de St. Vincent into no less than *fifteen ;* while the French Professor Broc has attempted to establish several *subgenera ;* and thus, as we think, he adduces decisive evidence of the facility wherewithal your fantastic visionaries can overleap the barriers of reason and nature. With the " affirmative opinion" on this speculation, ours is in perfect harmony; for, in the obvious proposition—that "the human species must have had one pair at least for their first parents" —and that the identity of organic structure and function, and all their essential elements, is complete in every diversity of the human kind—we recognize authority for considering the *unity* of the Human Race as a self-evident fact; a fundamental truth susceptible of perfect demonstration. Where it is proposed to distribute the Anthropic genus into two or more species, the proposition ought to be supported by the conclusive authority of distinct and manifest specific characters. Such a proposition as this however, as we have ever yet seen it, has clearly the aspect of a speculative assumption, conceived by fancy, propounded by vanity and unsustained by a single trace of probable testimony, analogical or inductive.

Dr. Morton notices the antropological systems of Linnæus, Buffon, Blumenbach and Cuvier, adding succinct remarks on their several merits. He admits the classification of Blumenbach to be "obviously imperfect;" but, for reasons of convenience, he adopts it for the ground-work of his introductory essay, resting himself content with an approximation to accuracy in this difficult department of natural science. Two leading features constitute the bases of most of the attempted classifications of the human species; and one of these methods is denominated the *physical*, the other the *ethnographic* system. In the former, mankind are grouped in great divisions characterised by similarity of exterior conformation; in the latter, the arrangement is based on analogies of language. Each of these systems has its advocates, to the exclusion of the other; but it seems reasonable, in Dr. M's. mind, to regard that method as the most natural and comprehensive which is derived from both these sources, as well as from all others which tend to establish analogies among men. In order therefore to combine, as far as possible, all these advantages, he proposes to consider the Human species as consisting of *Twenty-two* families. At the same time, he premises that these families are not assumed to be identical with *Races*, but merely as groups of nations possessing, to a greater or less extent, a resemblance of physical and moral character, and language—some of them retaining the peculiarities of the aboriginal races to which they belong, while others are of very diverse extraction and of comparatively recent origin. According to our philosophy, the Head affords by far the best "physical and moral"—we prefer the terms *organic* and *mental*—characters for distinguishing the different Races of men; and, as experience shows, these characters subsist with an extensive or general uniformity readily appreciable by disciplined observation. With other physiologists, we regard the head as a formation constructed by brain, skull and integumental tissues, and we recognize the *organic* characters in its shape and size, with the relative proportions of its parts, and their constitutional quality or temperament. We know, moreover, that the brain constitutes the primary mould from which the skull and head derive their configuration; and hence, it is our doctrine that the brain and skull are naturally adapted to be employed for the like purposes as the entire head, in a system of anthropological classification. After the same manner, we retrace the *Mental* characters through the shape, size and other conditions of the brain, as indicated by the peripheral forms of the head and skull. We may fairly contemplate the brain as an aggregate of organic instruments, and the Mind as an aggregate of powers or faculties; and, assured are we, that the cerebral instruments and mental faculties are co-existent and severally co-operative—each individual of the latter naturally using its own peculiar one of the former, in the exercise of its appropriate functions. Hence, on these principles, we may seek to trace the mental characters of disposition and capacity by inspection of the head, and the accuracy of the results is susceptible of trial by their correspondence with the mind's manifestations—in thought and feeling, discernible by reflection on consciousness; in speech and composition, in act and conduct, discernible by observation. We are desirous, in fine, of seeing the Head and its constituent organs adopted, in their forms, relations and conditions, as the source of elementary principles, in the anthropological and psychological systems.

Availing himself of Blumenbach's arrangement as respects the great divisions of mankind, Professor Morton constructs his system of anthropography on a distribution of the whole Human Species into five races and twenty-two families, with their numerous varieties. We follow his order and nomenclature, in exhibiting a concise view of his system.

I. THE CAUCASIAN RACE includes the Caucasian, Germanic, Celtic, Arabian, Libyan, Nilotic and Indostanic families. He represents this Race as being characterised by a naturally fair skin, susceptible of every tint; the hair fine, long and curling, and of various colours; the skull large and oval, with its anterior portion full and elevated; the face small in proportion to the head, of an oval form, with well-proportioned features; the nasal bones arched, the chin full, and the teeth vertical. The Caucasians are distinguished for the facility with which they attain the highest intellectual endowments.

II. THE MONGOLIAN RACE comprehends the Mongol-Tartar, Turkish, Chinese, Indo-Chinese and Polar families. The characters of this Race, are a sallow or olive-coloured skin, which appears to be drawn tight over the bones of the face; the hair long, black and straight; the beard thin; the nose broad and short; the eyes small, black, obliquely placed, with the eyebrows arched and linear; the lips turned out, the cheek-bones broad and flat, and the zygomatic arches salient; the skull oblong-oval, somewhat flattened at the sides, with a low forehead. In their intellectual character, the Mongolians are ingenious and imitative, and moderately capable of cultivation.

III. THE MALAY RACE embraces the Malay and Polynesian families. Its characters appear in a dark complexion, varying from a tawny hue to a very dark brown; the hair black, coarse and lank; the eyelids drawn obliquely upwards at the outer angles; the mouth and lips large; the nose short and apparently broken at its root; the face flat and expanded, with the upper jaw projecting, and the teeth salient; the skull high and squared or rounded, and the forehead low and broad. The Malayans are active and ingenious, and they possess all the habits of a migratory, predaceous and maritime people.

IV. THE AMERICAN RACE comprises the American and Toltecan families. Its characteristic marks are, a brown complexion, with long, black, lank hair, and a deficient beard; the eyes black and deep set; the brow low; the cheek-bones high; the nose large and aquiline; the mouth wide, the lips tumid and compressed; the skull small, wide between the parietal protuberances, prominent at the vertex, and flat at the occiput. The Americans (the ancient people and their descendants, he means) are naturally averse to cultivation and slow in acquiring knowledge, they are restless and revengeful, fond of war and wholly destitute of maritime adventure.

V. The Ethiopian Race contains the Negro, Caffrarian, Hottentot, Oceanic-Negro, Australian and Alforian families. It has for characters, a black complexion, with black woolly hair; the eyes large and prominent; the nose broad and flat; the lips thick, and the mouth wide; the head long and narrow; the cheek-bones prominent, the jaws projecting and the chin small. In disposition, the Negro is joyous, flexible and indolent; while the many nations which compose the Ethiopian race present a singular diversity of intellectual character, of which the far extreme is the lowest grade of humanity.

We will here introduce a passing remark on the Professor's anthropographical classification, and its elements. Instead of adopting the forehead as a chief "physical," and "intellectual" capacity as a principal "moral," character in the distinction of Races, he might have drawn three kinds of *organic* characters from the Head, by viewing it under a threefold distribution, corresponding with the three recognized lobes of the brain. From the Mind also, in a similar way, he might have obtained three kinds of *mental* characters, by considering its faculties under the threefold distribution into affections, sentiments, and intellectual powers. Such an arrangement would have yielded the principles of a consistent, though limited, scientific system; for, as in individuals, both families and races may be distinguished by the predominancy of one lobe of the brain over the other two, and of one order of the mind's faculties over the other two—for example, the anterior cerebral lobe may exhibit an ascendancy of size and energy over the other two, while the intellectual powers are displaying a high degree of vigour and activity over the two other orders of mental faculties. We find a clear illustration of this example in Dr. M.'s system; for, in "*the large oval head*" and its "*full elevated forehead*," co-existing with the *highest* "*intellectual*" *aptitude*, we distinctly perceive a remarkable confirmation of the psychosophical doctrine—that the anterior cerebral lobe, by its several parts, performs the functions of organic instruments, appropriated to the higher intellectual powers.

Conformably to the method preferred by him, Dr. Morton displays considerable ingenuity and research in using the physical and ethnographic materials wherewith he characterizes the numerous varieties, arranged under his twenty-two families of the Humankind. Notwithstanding many strong inducements to accompany him closely through this beautiful and highly interesting portion of his Essay, we feel reluctantly obliged to limit our observations to a few cursory notes, selected from his pages with a view to elucidate that natural correspondence which subsists between the shape of the head and the character of mind, in active life, which we regard physiologically as constituting the most important fundamental principle in Mental Science—not useless in education, government and legislation, in moral, religious and medical philosophy. We arrange our Notes numerically, adding the pages of Dr. M.'s volume with the object of facilitating reference.

I. *p*. 11.—Koordistan is inhabited by two sorts of people, the clansmen or tribesmen or military order, and the peasants or cultivators of the soil, and the difference in physiognomy between them is so perfectly distinguishable that it would be impossible for the latter to pass himself for his countrymen of nobler race. The peasant has a much softer and more regular countenance, sometimes showing the Grecian features: the tribesman is hard-featured, with a thick prominent forehead, abrupt lines, and grey or blue eyes usually fixed in a kind of stare : he holds the former in a state of absolute bondage. The Koords treat their women more kindly than the Turks or Persians, and have a better idea of domestic comfort: yet they are haughty and cruel, fond of war and pillage, and fight among themselves when they have no common enemy.

II. *p*. 12.—The unmixed Greeks are above the middle stature, having fine proportions and a graceful mien: the forehead is high, expanded and but little arched, so that it forms, with the straight and pointed nose, a nearly rectilinear outline: the face is a beautiful oval, and small in proportion to the voluminous head. The Roumelian population of Greece the most resembles her ancient inhabitants in moral traits: they are hardy, brave, and warlike, and have never been completely subjected by the Turkish aggressors. The voluminous head and lofty forehead co-exist universally with supreme intelligence; and, in the displays of this, the ancient Greeks have never yet been equalled.

III. *p*. 13.—The Roman head differs essentially from the Greek in having the forehead lower and more arched, the nose strongly aquiline with the nasal bones much depressed between the eyes. In the ancient busts and statues, we discern the genuine Roman type consisting in a large flat head, a low wide forehead, a face broad and square, with a short thick neck, and stout broad figure. Every page of the Roman history confirms the observation—that, associated with the large flat head and its low wide forehead, the character of persons and nations is distinguished by energetic manifestations of selfishness, pride, cruelty and stern perseverance.

IV. *p*. 13.—The Germans are well known for their middling stature, robust form, light hair, and fair florid complexion. In them, the head is large and spheroidal, the forehead broad and arched, the face round, the eyes blue, and the neck rather short. Their moral character is marked by decided personal courage, great endurance of fatigue, firmness and perseverance, with a strong attachment to their families and their native land. Intellectually, they are conspicuous for industry and success in the acquisition of knowledge. With a singular blending of taciturnity and enthusiasm, they rival all modern nations in music, poetry and the drama: nor are they less eminent for their critical attainments in language and the exact sciences.

V. *p*. 16.—The Celtic people possess strongly marked features. They are tall, athletic, little prone to obesity, and their physical strength corresponds to their muscular proportions. They have the head rather elongated, the forehead narrow and but slightly arched; their brow is low straight and bushy; the eyes and hair are light, the nose and mouth large, the cheekbones high, the face angular, and the expression harsh. They are slow but laborious, and endure fatigue beyond the sufferance of other men; in disposition, they are frank, generous and grateful, yet quick-tempered, pugnacious and brave to a proverb. We have the following most faithful graphic statement from the pen of M. Bory de St. Vincent, a very clever French philosopher. "The Celts and Gauls have become the modern French, of whom the Franks of the middle ages are not the parent stock, as those assert who trace their genealogy to the latter barbarians. It is from their Celtic ancestors that the French derive their vivacity, their inconstancy, their impetuous courage devoid of perseverance, a vanity often puerile, and a remarkable quickness of perception, together with that levity which is the jest of a neighbouring nation."

vi. *p.* 18, 19.—In the Arabian family, the face represents an elongated oval, with a delicately pointed chin and a high forehead; the eyes are large, dark and full of vivacity, the eye-brows finely arched, the nose narrow and gently aquiline, the lips thin and the mouth expressive. Some very opposite elements are blended in the moral character of the Arabs; they are the children of impulse; at one moment destroying or plundering the unresisting traveller; and the next receiving with open hospitality the stranger whose necessities have driven him to their tents. Except in their wars and pastimes, they are indolent; vanity is the characteristic of all classes; their covetousness and duplicity are remarkable; their politeness is extreme; their sobriety constitutes a national feature.

vii. *p.* 21.—Originally, the Hebrews or Jews were a pastoral people; but, in after-times, they occupied the cities and territory of Palestine, under a changeful sort of government by judges and kings. Since the extinction of their nation by the remorseless Romans, they have been ill-used outcasts in every land. Their physiognomy is familiar in the receding forehead, the elongated face, the large and aquiline nose. In its general bearing, the Hebrew head very faithfully indicates the mental proclivity of this most singular race. Whatever merits attach to their name for the display of high intelligence, these are due to individuals inspired with extraordinary energy in the exercise of extraordinary missions; but their devoted attachment to their religion and their patient endurance of adversity, are among the most striking traits of their character. Dispersed by a divine judgment, they are found almost every where on the habitable earth, recognized by the same features, the same obstinacy of purpose, and the same undeviating form of worship.

viii. *p.* 27.—On the ruins of their ancient monuments, and their mummies, we retrace the physical lineaments of the primitive Egyptians; and these remains commemorate two prominent varieties of this extraordinary family. There is a remarkable resemblance among the innumerable heads sculptured in the temples of the Nile; and one who is accustomed to examine them, becomes so familiar with the Egyptian physiognomy that when other races are introduced, as the Jews or Negroes, the eye can detect them without difficulty. There is also a singular accordance in conformation between the sculptured heads and the real ones taken from the Theban catacombs. One of the varieties is known by lowness and narrowness of the forehead: the other presents the full development of the Caucasian head: the former greatly predominates in the sculptures, and has been regarded conjecturally as characteristic of the Egyptian race. The nose was rather long, and joined the head much in the Grecian manner: the eye was elongated and rather oblique, the lips well formed, the chin rounded and moderately full, and the whole expression of countenance mild and pleasing; the hair was long, straight and generally black; and the person appears to have been spare, with long limbs and delicate hands and feet. The antiquity of the Egyptian nation, and their skill in the arts and sciences, have been proverbial in all ages; and it is a remarkable fact that the first glimpse we obtain of the Egyptian history and manners, shows a people already advanced in the arts of civilized life: the same customs and inventions that prevailed after the accession of their eighteenth dynasty, are found to have been established in the remote age of Osirtasen the cotemporary of Joseph. Their antiquity and learning are illustrated by the facts—they had completed the pyramids of Memphis within three hundred years after the era assigned to the Noachian deluge; they wrote their hieroglyphic characters on papyrus as early as the time of Cheops, two thousand years before Christ; they discovered and constructed the arch more than three thousand years ago: the Greek scroll is common in the tombs of the Pharaohs: and the so-called Doric column and entablature ornamented the porticoes of Beni-Hassan, before sculpture was an art in Greece: hence it is evident, that this singular family had attained a high degree of civilization and refinement at a time when the western world was still involved in barbarism, when the history of Europe had not yet begun, and long before Athens, Carthage and Rome were in existence. Physiological research will, one day, and that not a distant one, succeed in discovering facts sufficient to establish the conclusion that the "Learning of the Egyptians" was begun, continued and consummated by a race possessing the "full development of the Caucasian head."

IX. *p.* 32.—Among the Hindoos in general, the face is oval, the nose slightly aquiline, the mouth small, the teeth vertical and well-formed, the chin rounded and generally dimpled; the eyes are black, bright and expressive, the eye-lashes long, the brow thin and arched; the hair is long, black and glossy, the beard very thin. The Hindoo head is small and narrow, especially across the forehead which is only moderately elevated. By nature, the Hindoos appear to be a mild, sober and industrious race, warm in their attachments and fond of their children; but their love of the marvellous, fostered as it is by a fantastic religion, is almost without a parallel among nations. They are of a timid disposition and not inclined to cruelty; yet their avarice, which is extreme, leads them readily to commit murder for the most trifling acquisition. They practise deception with infinite art, to which falsehood and perjury form no obstacles. Their intellectual character is distinguished among the present Asiatic nations; but their learning has been very much devoted to comments on their sacred books, which are extremely numerous. They have had many admirable writers in poetry and the drama: they excel in some branches of mathematics, and especially in algebra: their antique architectural remains are of a stupendous kind, and consist chiefly of rock-hewn temples ornamented with elaborate sculpture. According to Bishop Heber's observations, the criminal calendar of India is generally full of gang-robberies, incendiarism, and analogous crimes perpetrated by native Hindoos, notwithstanding the apparent mildness of their manners; and the number of children who are decoyed aside, and murdered for the sake of their ornaments is dreadful. For all these horrors, the national system of religion is mainly answerable, inasmuch as whatever moral lessons their sacred books contain—and they are very few—are shut up from the mass of the people, while the direct tendency of their institutions is to evil. The Hindoo temper is decidedly good, gentle and kind. As a nation, the people are sober, industrious, affectionate to their relatives, faithful to their masters, easily attached by kindness and confidence; and, in case of the military oath, they are of admirable obedience, courage and fidelity in life and death. But their morality does not extend beyond the reach of positive obligations; and, where these do not exist, the Hindoos are oppressive, cruel, treacherous, and every thing that is bad. Thus, in a most remakable manner, the Bishop's dismally instructive observations corroborate those of Dr. Murray Paterson, published more than sixteen years ago in the Phrenological Transactions, and afterwards introduced into vol. II. of the Phrenological Journal, in an admirable article intituled an Essay on the coincidence between the natural talents and dispositions of Nations, and the development of their brains.

x. *p.* 43.—The modern Turks are of a middling stature, with an athletic form and well-proportioned limbs. Their head is round, their eyes dark and animated, and the whole face expressive and intelligent. In manner, they are proverbially courteous and taciturn; but their true character is marked by violence of passion, cruelty and vindictiveness. They are intelligent and ready in the acquisition of every kind of knowledge, and they would soon assume an elevated rank in literature, were it not for the trammels of superstition and fatalism.

xi. *p.* 44.—The Chinese are rather below the middle stature, stout-limbed and inclined to corpulency. Their head is large, rounded and somewhat conical, owing to a high retreating forehead. From inspection of specimens, the Chinese skull appears to be oblong-oval, in its general form: the frontal bone is narrow in proportion to the width of the face, and the vertex is prominent: the occiput is somewhat flattened: the orbits are of moderate dimensions and rounded, The Chinese eye is small, half closed and drawn obliquely upwards towards the temple; the upper eyelid projects a little beyond the lower; the eyebrows are black, highly arched and linear: the nose is small, flattened towards the nostrils, broad at its root, and separated from the forehead by a strongly marked depression. Contemplated in their moral character, the Chinese are distinguished among themselves by mildness and urbanity; by a wish to show that their conduct is reasonable, and generally a willingness to yield to what appears so; by docility, industry, subordination of the young, respect for the aged and for parents; and by their acknowledging the claims of poor kindred. These, however, are virtues of public opinion; and, of course, they have more show than reality, in particular cases; for, on the other hand, the Chinese are specious but insincere, jealous, envious and distrustful to a degree. Conscience for them has few checks but the laws of the land, and a little frigid ratiocination on the fitness of things which is generally ineffectual to restrain, when the selfish and vicious propensities of our nature may be indulged with impunity. The Chinese are selfish, cold-blooded and inhumane: in the punishment of criminals, by the practice of torture they are barbarously cruel: those in authority entirely disregard human suffering and human life, when the infliction of the one or the destruction of the other can be made subservient to the acquisition of wealth or power. Although, in letters, in science, and in art, the Chinese remain the same now what they were many centuries ago, yet their intellectual character is deserving of especial attention. That nation cannot be viewed with indifference which possessed an organized government, an army, a written language, historians and philosophers, coeval with the inspired Historian of the Creation. They have their national music and their national poetry; but, of sculpture, painting and architecture, they have no just conceptions; and their national pride prevents their adopting the arts of other countries. Their faculty of imitation is extraordinary, and their mechanical ingenuity is universally known to be pre-eminent. They possess a copious literature, both ancient and modern; they have known and practised the art of Printing, for eight hundred years; and their written language, with the same characters hitherto used, is of an extreme antiquity. Vessels of Chinese porcelain, not less than four thousand years old, have been discovered in the Egyptian catacombs, with inscriptions easily read by the scholar versant in Chinese philology. On three of these vessels is inscribed the legend—THE FLOWER OPENS, AND LO! ANOTHER YEAR. The Japanese bear a striking resemblance to the people of China, with the same features in an exaggerated degree. They are short, with heavy limbs, large heads and sunken eyes; and, like their "continental neighbours," they are laborious artificers, less ingenious, equally superstitious. For the psychological naturalist, it might be a legitimate and favourite inquiry—to determine demonstrably the constitution of mind and the co-existing configuration of head possessed by the people, who devised, enacted, and have perpetuated those social institutions which caused and continue the apoplexy of progressive civilization among so many of the oriental nations.

XII. *p*. 48.—The Aracanese are accustomed to flatten the heads of their children by means of a piece of lead, applied soon after birth; they slit and distend their ears to a frightful degree: and they are represented as being the most uncultivated and barbarous people of the Indo-Chinese family. By this detestable process of artificially flattening the forehead of infants, the head is deformed and the parts of its brain forcibly driven from their natural positions and relations; and, by necessary consequence, the head thus disfigured ceases entirely to be a natural subject of observation.

XIII. *p*. 48.—At its superior part, in the Siamese, the forehead is narrow; between the cheekbones, the face is broad; the chin is pointed, so that the whole contour seems rather lozenge-shaped than oval. The occipital portion of the head is nearly vertical; and, compared with the anterior and sincipital divisions, it is very small. The lateral halves of the head are not symmetrical: from the ear upwards, it rises to a great height, the posterior part of the coronal region being very prominent. Altogether, the Siamese head is peculiar: its diameter, from the front backwards, is uncommonly short, and hence the general form is somewhat cylindrical: and, in most instances, the occipital foramen is placed so far back that, from the crown to the nape of the neck, the line is nearly straight. Notwithstanding the Siamese are said to be remarkable for filial respect and regard for their rulers, yet the national moral character appears to be at a very low ebb.

They are described as being suspicious, vacillating and cruel; servile and cringing to their superiors, in the extreme; arrogant and tyrannical to those who are below them in rank. Their virtues and their vices are venal: the services of the judge and the assassin have each their price: sordid oppression and priestcraft, allied with wretchedness and filth, everywhere abound. An intelligent traveller regrets his not having met with one honest man in their land.

xiv. *p.* 49.—Among the inhabitants of Cochin-China, the general form of the face is round, so that the two diameters are nearly equal: the forehead is short and broad: the occipital portion of the head is elongated: they are a coarse featured people, and render themselves repulsive by the constant use of areca and betel, which reddens the lips and blackens the teeth. They are the gayest of the oriental nations; good-natured and polite, but extravagantly fond of etiquette. So versatile are their feelings and actions, that they have been compared to the monkey-race, whose attention is perpetually changing. They are active and warlike, but want industry and perseverance.

xv. *p.* 50.—In the Nicobar Islands, the natives compress the heads of newly-born infants, in such a manner as to flatten the occiput, and cause the teeth to project outwards. Their colour is a deep copper: they have thick lips and wide mouths. They live in a very uncivilized state and compel their women to cultivate the ground; hitherto, they have resisted all measures for the improvement of their condition.

xvi. *p.* 51.—Tbe Samoyedes are seldom more than five feet high: they seem "all of a heap," have short legs, small neck and a large head, with the nose and face flat, and the chin projecting. They are an indolent and savage people, and extremely apathetic on all those subjects that excite the feelings of other men: their women often become mothers at twelve years of age.

xvii. *p.* 56.—With the nose short, depressed and flattened; the eye small, black, oblique and expressive; the face broad, compressed and very prominent, the Malayan has a large head, with its forehead low, moderately full and arched, while the occiput is much compressed, and often projecting at its upper and lateral parts. The Malays possess an active and enterprising spirit; but, in temper, they are ferocious and vindictive: caprice and treachery are among their characteristic vices: and their habitual piracies are often conducted under the mask of peace and friendship.

xviii. *p.* 59.—With the Polynesian islanders, the stature is middling and athletic; the forehead low, but not receding; the eyes black, bright and expressive. Between the aristocratic and plebian classes however, there exists a great disparity as respects stature, features and complexion. The privileged order is much fairer and taller than the other: their heads are better developed, and their profile shows more regular features, including the arched and aquiline nose. The indolent habits of this caste dispose it to obesity which often becomes extreme after middle life. The Polynesians are intelligent, imitative, and docile; their progress in elementary literature and the more useful arts has been rapid; and perhaps no people on the globe has ever been more amenable to the usages of civilized life and the doctrines of Christianity. Their intellectual capacities have been considered equal to those of the Caucasian race: this much however is true of them—that, although they rapidly acquire ideas by means of their active perceptive powers, yet their reflective faculties have not hitherto expanded in proportion. In their uncivilized state, the Polynesians are singularly devoted to boxing, archery and boat-racing; but their most striking predilection is for maritime amusement and adventure. Their canoes are large, and constructed with great ingenuity: in these vessels they prosecute their wars, and undertake considerable voyages for profit or pleasure. The fondness of this people for the sea constitutes a national and predominating feature in their character: and, in their naval architecture, a high degree of ingenuity is conspicuous. —We should rejoice to meet with a clear and rational definition of the mental and organic agents, which co-induce and sustain this characteristic predilection for seamanship in the Polynesian mind.

XIX. *p.* 64.—The Appalachian head is rounded; the nose large, salient and aquiline; the whole face triangular; the chest broad; the body and limbs muscular. The nations composing this great branch of the American Family are warlike, cruel and unforgiving: they turn with aversion from the restraints of civilized life, and have made little progress in mental culture or the useful arts. The co-existence and co-efficiency of the round-head with a ruthless disposition may be contemplated and ascertained in the feline tribe, from the lion or tiger down to the domestic cat; and the study will be facilitated by instituting observations on the head or skull of a parlour cat placed side by side with that of a hare or rabbit, for the purposes of comparison.

XX. *p.* 65.—The physical aspect of the Fuegians is altogether repulsive, and their domestic usages are said to heighten the defects of nature. These islanders are of low stature; have large heads, broad faces and small eyes, capacious chests and clumsy bodies, with great knees and ill-shaped legs. Their expression of countenance is vacant: they seem destitute of the usual curiosity of savages, caring little for any thing that does not minister to their present wants. Dr. Morton attributes the difference between the Fuegians and other American nations to the effects of climate and locality, and the consequent habits of life, tending to depress and brutalize the mind, and to impair the physical man. This excessive degradation in the Fuegian character proceeds from the preponderance of animality in the constitution of their mind and the structure of their head, combined with a weight of the phlegmatic temperament. With a large head, having a lofty broad forehead and high intelligenee its inseparable associate, the dwellers in the dreary island of Tierra del Fuego would soon extricate themselves from the ungenial effects of its climate and locality.

XXI. *p.* 89.—The Caffers are tall, athletic and well-proportioned, with much natural grace of manner. Their head is large, with the forehead full and vaulted, the nose salient and aquiline, and the face a fair oval. Their physiognomy is remarkable in being a combination of the European and Negro features. This people is as much above the genuine Negro in morals and intelligence, as in physical appearance. The tribes resident near the English colony are less cruel and superstitious than some others; but their appeals to pretended sorcery in punishing crimes and in settling disputes, and the despotic sway of their chiefs, are taken for evidences of a great degree of barbarism.

XXII. *p.* 90.—One of the most singular varieties of the Human family is that of the Hottentots; their stature is of the mediate class; their persons are big and clumsy, with fine limbs, and remarkably small hands and feet. Their head is large, the forehead low and broad, the face extremely wide between the cheek-bones, whence it retreats rapidly into a contracted chin. The Hottentots have but very vague ideas of religious obligations, although they are prodigiously superstitious. They are inveterately indolent and gluttonous, devouring every kind of animal garbage that falls in their way, without preparation; and, when thus gorged, they throw themselves down and sleep off the effects. They are susceptible of some improvement however, and have been converted into tolerably efficient soldiers; hence, in the large head with its low broad forehead, the main traits of their character are not unfaithfully indicated.

XXIII. *p.* 91.—Throughout the Indian Archipelago and in many islands of the Pacific ocean, the various peoples, collectively denominated Papuas, are extensively dispersed. They are easily recognized as members of the great Negro race, by their similarity of organic features and mental capacities. In them, the head is rounded, yet compressed in front and at the sides, with the forehead greatly depressed: their manners are savage, and they exhibit little aptitude for moral and intellectual improvement.

XXIV. *p.* 93.—The New Hollanders are of the full stature, with ample chests and thin bodies, with long slender limbs. Their face is frightfully ugly: it projects greatly from the head: the mouth is particularly prominent owing to its width and the great size of the lips: the nose is flat and broad, with the nostrils expanded: it is separated from the forehead by a deep sinus. The frontal ridges often overhang the eyes, while the forehead itself is low and slopes rapidly to the top of the head. They have fierce and vindictive tempers, and are passionately fond of war: they are perpetually embroiled in feud and bloodshed: even their courtship consists in a violent abduction of the object of their desire: through life, their women are treated with unparalleled cruelty. They are most disgustingly filthy in their persons, and gluttonous in their eating; their dances betray the licentiousness of their morals; they are all but incapable of civilization.

We have embodied these gleanings of "physical and moral" description as elements of an anthropological system wherein the distinction of Races might be grounded on essential differences in configuration of the head and co-existent essential differences in constitution of the mind. Although as sketches, they are necessarily imperfect, yet, in being recorded on the pages of this journal, they will remain accessible to the investigation of scientific naturalists. Dr. Morton deserves high commendation for care and faithfulness in constantly referring to the sources of his information and authority; and, while admiring his method in thus honourably citing these observations, we would persuade all travellers to note such differences with minute attention, and thus to simplify and increase the first principles of Psychology.

From Dr. M's. general observations on the barbarous nations composing the American Family, p. 65, we derive much new and most valuable insight into the peculiarities of organization and mind which characterize this remarkable people. In retracing these peculiarities, we limit our selections to his distinctions of national heads, and of national minds as manifested in modes of life and social institutions. After examining a great number of skulls, Dr. Morton finds that the nations east of the Alleghany mountains, together with the cognate tribes, have the head more elongated than any other Americans; he applies this remark especially to the great Lenape stock, the Iroquois, and the Cherokees. To the west of the Mississippi, the elongated head again occurs in the Mandans, Ricaras, Assinaboins, and some other tribes. Yet, even in these instances, the characteristic truncation of the occiput is more or less obvious, while many nations east of the Rocky Mountains have the rounded head so distinctive of the race—as the Osages, Ottoes, Missouris, Dacotas and numerous others. In Florida, the same conformation of head is common; but, as Dr. M. observes, some of these nations are evidently of the Toltecan Family, as both their characters and traditions testify. The head of the Charibs, as well of the Antilles as of Terra Firma, is also naturally rounded; and we may trace this configuration through the nations east of the Andes, the Patagonians, and the Chilian tribes. Flatness of the occipital portion of the Cranium probably characterizes a greater or less number of individuals in every tribe, from Tierra del Fuego to the Canadas. When these skulls are viewed from behind, their occipital outline is observed to be moderately curved outwards, wide at the occipital protuberances, and full from those points to the opening of the ear: from the parietal protuberances, there is a slightly curved slope to the vertex, producing a conical or rather a wedge shaped outline. As in this, there is no race on the globe, as Humboldt has observed, in whom the frontal bone is so much depressed, and the forehead is so small; but with Dr. M. we must admit that the lowness of the forehead is, in some measure, compensated by its breadth, which is generally considerable. Among a vast number of tribes, the flat forehead was esteemed beautiful; and this fancy has been the principal incentive to the moulding of the head by art. In many, the eyes are deeply set in the head; an appearance which is much increased by the low and prominent frontal ridges.

Dr. Morton divides the unsophisticated Americans into three great classes—those of Hunting, Fishing, and Agriculture, the pursuits on which they depend for subsistence. The first embraces most of the strictly nomadic tribes and a great proportion of the entire race. Those who on fishing subsist exclusively are not numerous. The natives of Tierra del Fuego, and the Flathead septs on the Columbia river, are among the proper piscatory tribes. Many nations inhabiting the Atlantic coast make their means of support a secondary consideration; some alternating it with agriculture, others with the chase. Many islanders, unacquainted with agriculture, are sustained for a great part of the year by fishing in the rivers and lakes; and, in the interval between the ending and recommencement of the fishing season, they are driven to the greatest extremities for food. Thus, the Shoshones west of the Rocky Mountains live more than half the year on roots alone; and the Ottomacs on the Orinoco are compelled for months together to assuage the cravings of nature by mixing with their food a large proportion of unctuous clay. It is remarkable that these piscatory people evince no fondness for the sea: they appear to be entirely destitute of inclination and spirit for maritime adventure. Before the arrival of the Europeans, a few of the American tribes were strictly agricultural: since that event, a much greater number have adopted the same mode of life. Among the former, are the lowlanders who occupy the plains and open land between the Orinoco and the Amazon, in the south, and in the north, those who dwell between the great lakes and the Gulf of Mexico, and between the Mississippi and the ocean. Even with the most industrious of those tribes however, their agriculture was confined to the cultivation of Indian corn, the sweet potatoe, tobacco and melons. From the force of example, the Cherokees have become an agricultural nation, but, in Mexico, there are tribes who have led the peaceable life of cultivators of the soil, for some centuries, exempt from the contingencies to which the hunters are exposed. Many of these nations resort to the three modes of subsistence, employing the spring in fishing, the summer in agriculture, and the autumn and winter in hunting. Although the American originally obtained the horse through the European intruders; yet, from the first, he has managed this noble animal with surprizing dexterity. Strange it is however, that there scarcely exists an example, among the free Indians, of a horse being used for agricultural purposes.

Regarding the moral nature, or rather the mental manifestations, in the American savage, Dr. Morton's observations are distinguished by the best and strongest features of research, reflection and experience. He recognizes in the race, a boldness of physical development accompanied by a corresponding acuteness in the organs of sense; it is his opinion that, although Nature has done much in producing this acuteness, yet Education has contributed more to its perfection. We would allow ourselves to be led to the somewhat different induction, with reference to the Americans—that, in perfecting the sentient faculties and their organs, Education does much by increasing the quickness and range of their action, but Nature does much more by implanting great innate aptitude for instruction in the senses and great inherent energy in the organs through which these faculties execute their functions. With Dr. M. we reject the idle theory which attributes to the American Race, in comparison with the European, a physical inferiority, less hardiness of constitution, lower powers of endurance. By day and by night, in summer and winter, over mountains and marshes, through rivers and forests, the Indian will pursue his determined course, whether his object be revenge on an enemy or food for his family at home. Human effort never exceeded his, in the patient sufferance of fatigue and hunger, of thirst and cold: but, he is incapable of servitude; under enslavement, his spirit sinks; and, with its fall, his physical energy perishes.

Dr. Morton has successfully studied the Americans; and he defines their character with much fairness; we take notes from his delineations. He represents cautiousness and cunning as its most prominent features, with a systematic vigilance which marks every action: their very politeness is a part of this cautiousness: they never express themselves with surprise. When an object interests an Indian by its novelty, he shews his gratification in a few subdued remarks or by a significant gesture: but it is difficult to betray him into enthusiasm. That taciturnity which is fostered by all his national usages, has its source in a concentrated cautiousness: this even appears in the marriage-ceremony which is often joyless and melancholy, as if it were rather the harbinger of sorrow than of happiness. Seldom do the pastimes of this people excite hilarity or heated feeling, unless the performers are stimulated by intoxicating drinks; in which case, as among more civilized men, a temporary madness unmasks the darkest passions, and the natural reserve of the Indian gives place to extravagant mirth and the most brutal ferocity.

Courage and fortitude form strong traits in the character of an Indian; but he is remarkable for improvidence, the result of habitual indolence. It is usual to charge him with treachery; but, in most instances, he has only retorted the perfidiousness that has been heaped upon him by others: he has retaliated on the oppressors, who practise every iniquity to deprive him of liberty and life, who desire the extermination of his race. He is accused of inhospitality; but, as concerns this imputation, Dr. Morton becomes his apologist, and clears him of the contumely. Covetousness does not predominate in the character of an Indian: he is singularly content with the supply of present need: his mind is rarely harassed with the idea of future want: he craves not the house or the land of his neighbour, and shows an entire apathy to those possessions which are most prized by civilized communities. Hence it has come, that the tumuli of Mexico and Peru, though immensely rich in gold and silver, were never disturbed by the native inhabitants: the sepulchres of their fathers were desecrated and plundered by the sacrilegious hands of strangers.

At home the Indian is least to be admired: there, he is lazy, vicious and sensual; habitually cold to the gentler sex, and stern to his children; a drone and a despot. He is less swayed by superstitious fears than most other savages; and his religion is more distinguished for its poverty than its grossness: it is chiefly a simple theism, which acknowledges a good spirit who exerts a benign influence on the destinies of men, and an evil spirit who is held to be the author of all misfortunes. An untutored American observes no regularity in the time or manner of his worship: this is the mere result of occasion or impulse. He hears God in the winds and the cataract: he acknowledges the divine presence in all the appearances of nature, attributing them to One spirit not to a multiplicity of spiritual agents. He is not addicted to idolatry: he believes in the immortality of the soul, which is to enjoy in a future state the most exciting temporal pleasures—the pastimes of hunting and fishing—without fatigue or alloy. They cherish an extraordinary veneration for the dead; and this sometimes induces them, on removing from one part of the country to another, to disinter the remains of their deceased relations, and bear them to the new home of the tribe.

When compared with those of the Caucasian and Mongolian Races, the Americans appear to be of a decidedly inferior cast. They are not only averse to the restraints of education; but, for the most part, they are incapable of a continued process of reasoning on abstract subjects: their minds seize on simple truths with avidity, while they at once reject whatever requires investigation and analysis. As for their mode of thinking, manner of life, and social condition, they have kept these nearly unchanged since the primitive epoch of their existence. They have improved but little in the building of their houses and boats; their inventive and imitative faculties are of a very humble grade; they are destitute of all predilection for the arts or sciences; they experience great difficulty in comprehending any thing that belongs to numerical relations. However much the philanthropist may lament over the fact, it nevertheless is an established fact—that for thousands of years, the Indian mind has continued incapable of civilization.

Under the form of a foot-note, p. 29, Dr. Morton introduces a concise but perfectly satisfactory disquisition on the supposed affinity between the Egyptians and the Negroes. In this, by pure induction from architectural, sculptural, pictorial, historical and other evidences, he arrives at the conclusion—that the ancient Egyptians is absolutely different and naturally superior, in morals and intelligence, to the Negro race. With reference to the physical character of the Egyptian, there is a source of evidence which he appreciates more highly than any other—he refers to the embalmed bodies from the Theban catacombs. These vast cemeteries were crowded with genuine Egyptians, whose remains even now retain almost every feature in perfection. Here are the very people who walked the streets of Thebes, with them who built Luxor and the Pyramids; and yet, among the thousands whose bodies the unholy hands of curiosity and avarice have dragged from their tombs, not one solitary Negro has been discovered. We admire this note as an eloquent and erudite contribution to the natural history of Man.

Another note of Dr. Morton's, p. 88, conveys the following observations. So certain is the great antiquity of the Negro race, as to have induced more than one philosopher to indulge in the conjectures—that this was the primitive stock of mankind, and that all the other varieties were derived from it alone, by the action of physical causes. According to accredited dates however, and in the words of a distinguished author, it is four thousand one hundred and eighty-nine years since Noah and his family came out of the ark. They are believed to have been of the Caucasian race; and since there is no ground for questioning the correctness of this belief, let it be assumed as a truth. Now, three thousand, four hundred and fifty-five years ago, a nation of Ethiopians is known to have existed: their skins were dark, and they differed widely from Caucasians in many other particulars: they migrated from a remote country, and took up their residence in the neighbourhood of Egypt. Supposing that people to have been descendents of Noah, their change must have been completed, and a new race formed, in seven hundred and thirty-four years, and probably in a much shorter period. According to Dr. Morton's views, this statement receives additional force from the recent discoveries in Egypt in as much as they show beyond all question that the Caucasian and Negro races were as perfectly distinct, in that country, upwards of three thousand years ago as they are now. Hence, he regards it as evident—that, if the Caucasian was derived from the Negro, or the Negro from the Caucasian, by the action of external causes, the change must have been effected in at most one thousand years: a theory which the subsequent evidence of thirty centuries proves to be a physical impossibility; and, for this reason, he insists that such a commutation could be produced by nothing short of a miracle.—Relatively to this question, our anthropological hypothesis involves the three-fold proposition—that Noah and his family were of an Antediluvian race superior perhaps to the Caucasian, in organic and mental endowment; that the three primary Races originated in the offspring of his sons; and that the foundation of distinct Families was then laid, their distinctions being subsequently completed and varied by the action of external, physical and social causes combined.

With feelings of unusual satisfaction with his matter and manner, his spirit and principles, as displayed in the Introductory Essay, we now enter with Dr. Morton on the field of skulls—his American Cranioscopy, with its exquisite graphic, and its luminous descriptive, illustrations. He begins, and we follow him, with the figures of Ancient Peruvian skulls, preceding them with the representations of an embalmed head, disinterred from a cemetery at Arica, and obviously a relic of antiquity. This head " has not all the characters of the Ancient Peruvian," nor does Dr. M. introduce it as " an unequivocal example of that race." With evident accuracy, he describes the forehead as extremely retreating, and partially moulded by artificial means; but the whole cranium is broader, both in its frontal and parietal diameters, than is common in the forementioned people. The sharpness of the superciliary ridges indicates the effects of a broad plate or bandage which has compressed the frontal bone and widened the entire head; apparently, it belonged to a person of distinction.

On the second plate, is figured the skull of a child not more than five years of age. Here, we are struck with the greatly inclined forehead, the extreme elongation of the whole head, and more particularly with the length of the occiput behind the ear; yet there is but little expansion of the head which, with the face, is narrow in proportion throughout. The third figure is that of a skull with a singularly flat and retreating forehead, and projecting face: the head, however, is not remarkable for narrowness; and, if any, a very slight degree of pressure has been applied to the frontal bone. This is the cranium of a woman about thirty years old: her entire desiccated body was obtained from the Arican district, with the hair very long, and retaining its natural black colour. With her, were found some pieces of an aromatic gum, and a small bag, containing some copper fish-hooks and small instruments of bone which were probably used in forming the meshes of nets or other fabrics. Five of the six skulls, discovered near the lake Titicaca, are strikingly like this female's, both as respects their general form, their narrow face, their small size, and their several diameters: yet they present more obvious marks of artificial modification.

Dr. Morton regards the relic, figured on the fourth plate, as furnishing an example of the head of the primitive Peruvians; and therefore, he takes it for a type of the cranial conformation of this people. Though the forehead retreats rapidly, there is but little expansion at the sides; and, from the face to the occiput inclusive, there is a narrowness that seems characteristic of the Race. Prefixed to the explanatory text, Dr. M. gives reduced outlines of his subjects: in this instance, the posterior view represents the skull elevated in that region without any unnatural width at the sides, and the vertical view sufficiently confirms the latter fact. This skull was found, about a mile from Arica, in a cemetery of the " ancient Peruvians." The body to which it pertained was placed in a squatting posture, with the knees drawn up and the hands applied to the sides of the head: the whole was enveloped in a coarse but close fabric, with stripes of red which had wonderfully withstood the destroying effects of ages.

On the fifth plate, there is a cranium strikingly analogous to the three preceding specimens, but the professor had not ascertained from what particular part of Peru the relic was procured. The intervention of art, in flattening this skull, is very manifest; yet the depression has been effected on a forehead extremely low by nature—for the lateral swell is not remarkable; and, in particular, the parietal protuberances are not much more inflated than those of the skull, previously set down as " a type of the cranial conformation of these people." Dr. M. concludes his section headed

"*The Ancient Peruvians,*" with stating summarily—that the average internal capacity* of the Caucasian or European head is at least ninety cubic inches, and it will be observed, he thinks, that the three adult skulls, in the preceding series of ancient Peruvians, give an aggregate of two hundred and nineteen cubic inches, or a mean of seventy-three only. It will also be observed, he says, that the mean capacity of the anterior chamber is about one half of that of the posterior, or twenty-five to forty-seven; while the mean of the facial angle is but sixty-seven. Hence then, on the evidences afforded by this statement, by the results of Dr. M's. inestimable table of anatomical measurements, and by the universal physiological principle, that *size* of the brain is a chief measure of mental *power* or energy, we readily arrive at the induction—that these "Ancient Peruvian" skulls, taken as the type of a family must have pertained to a nation naturally much inferior in mental power to the Caucasian and Mongolian Races: in fact the people, whose skulls included brains of this shape and size, never could display a high moral and intellectual character: such heads never excelled in the arts nor did they ever earn the homage intuitively yielded, by civilized minds, to eminence in the sciences.

Before entering on his inquiry into the physical and intellectual character of the Ancient Peruvians, Dr. Morton premises some descriptive and explanatory remarks: from these we extract a condensed selection. Peru, he says, is a narrow strip of land between the Andes and the sea, bounded on the south by a desert. It possesses many natural advantages, in its climate, soil and situation, and these appear to have been fully appreciated by its aboriginal occupants; for, he adds, there is evidence that several populous nations held successive dominion in the country. Even before the advent of the Spaniards, history throws much light on one of these nations—that which was governed by the Incas. With respect to the others, little else is known than what can be gleaned from their monuments and cemeteries. Atacama, an arid region dividing Peru from Chili, was the favourite sepulchre of the Peruvian tribes for many ages; for, the doctor observes, while the climate tends rather to the desiccation than to the decay of the dead, the sand and salt of the desert have contributed to the same end; and, in consequence, the lifeless bodies of whole generations of the former inhabitants of Peru may now be examined, like those from the Theban catacombs, after the lapse of centuries, perhaps of thousands of years. The great number of desiccated bodies remaining in these regions, serves to convey an idea of the vast population that has, at different periods, derived its subsistence from that country. From an examination of nearly one hundred Peruvian skulls, in his own and other collections, Dr. Morton deduces the result—that Peru has apparently been peopled, at different periods, by two nations with Crania differently formed; and, he thinks, one of these is extinct, or

---

* Dr. Morton gives the precise measurements, both of capacity and size, for each individual subject, throughout his work; and, at p. 257, he adds an elaborate and most valuable "Table of Anatomical Measurements," accompanied by another exhibiting their mean results. He is also particularly careful in explaining his mode of taking these measurements, with descriptions of the instruments and processes he employed; they are altogether original and ingenious, and happily calculated to attain the desired ends.

at least exists only as blended by adventitious circumstances in various remote and scattered tribes of the present Indian race. That family which preceded the Incas, he designates the Ancient Peruvian of which the remains have hitherto been found only in Peru and especially in its Bolivian division. Their tombs abound on the shores and islands of the great Lake Titicaca, in the interalpine valley of the Desaguadera, and in the elevated valleys of the Peruvian Andes. Collao was the name of the country around this inland sea, and Tiaguanaco appears to have been the site of its chief city.

Dr. Morton institutes an exposition of the physical and intellectual character, the history and tradition of the Ancient Peruvians, and he grounds it on what information he could glean from their monuments and cemeteries. However meagre his facts may appear, he thinks they possess considerable interest, and the more so because few others are available. He admits that all his knowledge of their physical appearances is derived solely from their tombs—a necessarily imperfect source of information. This people, he says, appears to have been in no respect remarkable in stature: neither, except in the conformation of the head, did they differ from the cognate nations: in this, he finds no evidence of mechanical compression, but is satisfied that it appears to be of the natural form, unaltered by art. Altogether, the Ancient Peruvian head is small, greatly elongated, and narrow in its whole length, with a very retreating forehead: but, he states, it possesses more symmetry than is usual in skulls of the American race. The face projects, the upper jaw is thrust forward, and the teeth are inclined outward: the orbits of the eyes are large and rounded, the nasal bones are salient, the zygomatic arches expanded, and there is a particular simplicity in the sutures that connect the cranial bones.

Dr. Morton foresees the very probable supposition, " that a people with heads so small, and so badly formed would occupy the lowest scale of human intelligence." Such however was not the case, as he says; and he proceeds " to show that civilization existed in Peru anterior to the advent of the Incas, and that those anciently civilized people constituted the identical nation whose extraordinary skulls are the subject of his present inquiry." Now, since the doctor himself appears to feel that, in this instance, his position is wonderful or dubious—made sufficiently dubious by evidence on most pages and plates in his book, and its table of anatomical measurements —we will essay to exhibit the grounds of his opinion under a distinct and conspicuous arrangement.

I. Among the first European travellers in Peru, was Pedro de Cieca, an officer in Pizarro's army: and, although an unlettered man, he describes with simplicity and clearness whatever came under his observation. His chronicle *

* Pedro de Cieca: Chronica del Peru, Cap. cv.; 18vo. Anvers, 1554. This curious book was translated into English, with the title, The Seventeen Years' Travel of Peter de Cieca, through the mighty kingdom of Peru, and the large provinces of Carthagena and Popayon in South America; by John Stevens; 4to. London, 1709. There was also an Italian version; 12mo. Venezia, 1560. Dr. Morton likewise refers to Acosta; Historia de las Indias, Lib. VI. Cap. 14; 4to. Sevilla, 1590. Acosta's Natural History was translated into French, German, Italian, and English, by Edward Gremestone; 4to, London, 1604.

contains the following passage; and after Dr. M. we give it entire.—"Tiaguanico," says Pedro, "is not a very large town, but it is deserving of notice on account of the great edifices which are still to be seen in it. Near the principal of these, is an artificial hill raised on a ground-work of stone. Beyond this hill, are two stone idols resembling the human figure, and apparently formed by skilful artificers. They are of somewhat gigantic size, and appear clothed in long vestments, differing from those now worn by the natives of those provinces: and their heads are also ornamented. Near these statues, is an edifice which, on account of its antiquity and the absence of letters, leaves us in ignorance of the people who constructed it; and such indeed has been the lapse of time since its erection, that little remains but a well-built wall, which must have been there for ages, for the stones are very much worn and crumbled. In this place also, there are stones so large and so overgrown that our wonder is excited to comprehend how the power of man could have placed them where we see them. Many of these stones are variously wrought; and, some having the form of men, must have been their idols. Near the wall, are many caves and excavations under the earth; but, in another place more to the west, are other and greater monuments, consisting of large gateways and their hinges, platforms and porches, each of a single stone. What most surprized me while engaged in examining and recording these things, was that the forementioned gateways were formed on other great masses of stone, some of which were thirty feet long, fifteen wide, and six thick. Nor can I conceive with what tools these stones were hewn out; for it is obvious that, before they were wrought and brought to perfection, they must have been vastly larger than we now see them. Before I proceed to a further account of Tiaguanico, I must remark that this monument is the most ancient in Peru; for it is supposed that some of these structures *were built long before the dominion of the Incas,* and I have heard the Indians affirm that these sovereigns constructed their great buildings in Cuzco after the plan of the walls of Tiaguanico; and, they add, that the first Incas were accustomed to hold their court in this place. Another very curious fact is, that in the greater part of this territory there are no quarries nor rocks whence the materials for these structures could have been derived. In the presence of Juan de Varagas who commands here, we asked if these edifices were built in the time of the Incas? But they laughed at the question, adding that they did not know who built them, but that they had a tradition of their ancestors, that these structures appeared in a single night." These statements and many others to the same purpose, Dr. M. adds, are confirmed by Diego de Alcobaza, the vicar-general, who also visited Tiaguanico and has left an account* of the wonder he saw at that place.

II. It will be observed by the preceding narration, Dr. M. goes on to say, that tradition among the Peruvians attributed these Cyclopian structures to an era long antecedent to the appearance of the Incas, and this tradition is sustained by history; for the city of Tiaguanico did not fall into the hands of the Incas until the reign of Mayta Yupanque the fourth king, at which period the edifices in question must have been in existence for centuries, and were already in a state of ruin and decay. Garcilaso de la Vega, himself of the royal Peruvian family, admits that these ruins existed at the time the country was conquered by his ancestors; and a Peruvian author, two centuries and a half nearer our own time, states (Mercurio Peruvians, Lima, 1791,) that Tiaguanico is indisputably anterior to the monarchy of the Incas, and speaks, as if from personal observation, of a gigantic pyramid and colossal human figures cut from solid rock, in-

---

* Garcilaso de la Vega: Commentarios Reales, que tratan del origen de los Yncas, reyes que fueron del Peru, de su idoltria, leyes, &c. con la historia general del Peru; folio, 2 tomes, Lisboa y Cordova, 1609—17.

dicative of the power and genius of a great nation. The first invasion of the Incas was followed by the erection of some temples to enforce the new religion, but their only great architectural monument in these parts, the Temple of the Sun on the island of Titicaca, was not built until the reign of Tapac Yupanque the tenth Inca, early in the fifteenth century. Herrera also alludes to a tradition of the Indians that these edifices have been built by Amazons at a remote era, nor are the Incas mentioned as having any part in their construction. Humboldt says (*Monuments*, I. p. 5), "it is probable that the edifices called in Peru by the name of *Inga-pilca*, or Buildings of the Inca, do not date farther back than the thirteenth century. Those of Vinaque and Tiaguanico were constructed at a more remote period: so also were the walls of unbaked brick, which were made by the ancient inhabitants of Quito. It is to be desired that some intelligent traveller would visit the banks of the great lake Titicaca, the province of Collao, and more especially the elevated plain of Tiaguanico, which is the centre of an ancient civilization in this region." Dr. M'Culloch (*Researches*, p. 406) remarks, in confirmation, "that a certain degree of demicivilization prevailed in the nations adjoining the Peruvian empire, which was not derived from their communication with the latter."

III. It will now be asked, in Dr. M's. words, "What evidence can be adduced to prove that the people, whose remains we are considering, were the same with those who have left the architectural monuments of Tiaguanico and Titicaca?" He regards the fact as established by the observations of Mr. Pentland,* an Englishman who had recently visited the upper Peruvian provinces. He states that in the vicinity of Titicaca, he had "discovered innumerable tombs, hundreds of which he entered and examined. These monuments are of a grand species of design and architecture, resembling Cyclopean remains, and not unworthy of the arts of ancient Greece or Rome. They therefore betokened a high condition of civilization: but the most extraordinary fact belonging to them is their invariably containing the mortal remains of a race of men, of all ages, from the earliest infancy to maturity and old age, the formation of whose crania seems to prove that they are an extinct race of natives who inhabited upper Peru above a thousand years ago, and differing from any mortals now inhabiting our globe. Specimens of their skulls are now in London and Paris: they are remarkable for the extreme extent behind the occipital foramen; for two thirds of the weight of the cerebral mass must have been deposited in this wonderfully elongated posterior chamber: and, as the bones of the face were also much elongated, the general appearance must have been rather that of some monkey family than of human beings. In the tombs, as in Egypt, parcels of grain were left beside the dead; and, it was another singular circumstance, that the maize or Indian corn so left, was different from any that now exists in the country." Mr. Pentland expresses his decided opinion, "that the extraordinary forms thus brought to light of day, after their long sojourn, could not be attributed to pressure or any external force, similar to that still employed by many American tribes; and, in confirmation of this view he adduces the opinions of Cuvier, Gall, and of many other naturalists and anatomists. On these grounds, he is of opinion that they constituted the population of these elevated regions, before the arrival of the present Indian population which, in its physical and moral characters, offers many analogies with the Asiatic population of the old world."

IV. As the Professor thinks, "the preceding facts appear to establish two important propositions: *first*, that the primitive Peruvians had attained to a considerable degree of civilization and refinement, so far at least as architecture and sculpture may be adduced in evidence, long before the Incas appeared in their

* Report of the Fourth Meeting of the British Association, p. 624; and Waldie's Journal of Belles Letters, for 1834.

country: and *secondly*, that these primitive Peruvians were the same people whose elongated and seemingly brutalized crania now arrest our attention; and, it remains to inquire whether these are the same people whom the Incas found in possession of Peru, or whether their nation and power were already extinct at that period." We should rather have preferred the inquiry, Whether the people, with elongated and brutal skulls, were the *Sculptors* of stone idols, resembling the human figure, gigantic in size and clothed in long vestments, and of colossal human figures cut from solid rock, and the *Architects* of enormous gateways with their hinges, platforms and porches, of a gigantic pyramid, and of innumerable tombs of a grand species of design and architecture, resembling Cyclopean remains and not unworthy of the ancient Grecian and Roman artists? But we must accompany the Professor to the completement of his inquiry.

V. Entering on it, he says—the modern Peruvian empire had existed upwards of four hundred years at the time of the Spanish conquest, so that its origin may be dated somewhere about the year MC. of our era. Now it appears, he argues, that among the first military enterprizes of this new family, was the conquest of Collao, which possessed a productive soil and a warlike population, and embraced within its confines the Lake Titicaca from which the Incas pretended to have derived a supernatural origin. Every effort was therefore made to subdue and destroy the Collas. The Inca Yupanque waged against them a war of extermination; and, we are told by Herrera (*Historia de las Indias*, Dec. III. Lib. IX, c. 4.) that, "in some of the towns, he left so few persons alive that inhabitants were afterwards sent from other parts of Peru to colonize the wasted districts: that in order further to depopulate the country, the natives were banished from it in large bodies, and dispersed through other provinces of the empire: and yet such was the dread in which the new dynasty held these warlike people, that they forbade more than a thousand of them to be within the walls of Cuzco at a time, lest they should attempt some revolutionary enterprize. It therefore appears that no means were left untried to subdue and exterminate the people of Collao. Yet the Doctor adds confessingly, how far such a system persisted in at intervals for more than two centuries, could have annihilated a whole nation, I shall not attempt to decide.

VI. When the Spaniards took possession of these provinces, they found them inhabited by barbarous tribes; and the islands of the Lake Titicaca, which had once been highly cultivated, were then waste and vacant. Upon the lake, were seen rafts made of the reed called by the natives *totora*, and on these rafts whole families made their home, tossed here and there upon the waters by every change of wind. They were in so brutalized a state that when asked to what nation they belonged, they replied! "We are not men, but Uros," as if they did not consider themselves as belonging to the human species. Were these Uros, the professor inquires, the remains of the savage colonies sent from other parts of Peru to supplant the Collas? this inference bears at least the stamp of probability: but, he admits, it still does not aid us in ascertaining whether the Collas themselves were the remains of the primitive civilized Peruvians.

VII. Garcilaso describes the Peruvian tribes near the sea-coasts, to whom he applies the collective name of Yuncas, as living in the utmost barbarism at the advent of the Incas. In proof of this statement, he adduces their mythology, which accorded divine attributes to every thing in which they observed any dominant excellence. Thus, says he, they worshipped the fox for his cunning, the deer for his swiftness, and the eagle for the perfection of his sight. These superstitions, however, are not more surprising than those of the primitive ages of civilization in the old world; and there appears throughout the Spanish historian an evident disposition to depreciate the character of the ancient tribes, in order to palliate the cruel measures which were resorted to by the Incas for their subjugation. Garcilaso himself describes a remarkable temple at Pachacamac which was erected by the Yuncas; and the Chimuyans, who were something farther to the south, appear to have possessed extensive and regular edifices, together with some other attributes of civilization. The inhabitants of Chimu resisted the Incas with great valour, and appear to have been very superior to most of the adjacent tribes, at that early period. Nevertheless, they could not compare with the primitive nation of Collao; and, when we find the remains of the latter mingled, as it were, among those of the barbarous hordes on the sea-coast, their presence may be accounted for in the casualties of war or commerce, or by that forced system of colonization which has already been described. Thus Dr. M. concludes, have I followed the researches of Baron Humboldt and Dr. M'Culloch with the more zeal, because so little notice has been taken of the subject by other writers, and especially because we are now able to take one step more in the inquiry, by studying the arts of these people in connection with their cranial remains.

VIII. Dr. Morton closes his inquiry with the announcement, that Mr. Stevenson (*Travels in S. America, II.* 22, 170, 173) has described very interesting remains near the village of Langunilla, in the province of Caxamarca, which he supposes to be anterior to the Inca dominion in Peru. He represents these remains to be those of a town, of which the houses are all built of stone surrounding a rock or hill in a valley. The bottom range of rooms in them has walls of an amazing thickness, containing stones twelve feet long and seven feet high, forming the whole side of a room, with one or more large stones laid across, which serve as a roof. Above these houses, another tier was built in the same manner, on the back of which were the entrances or doorways, and a second row had their backs to the mountain. The roofs of the second tier in front had been covered with stone, and probably formed a promenade; a second tier of rooms thus rested on the roofs of the first, which were on a level with the second front tier. In this manner, one double tier of dwelling-rooms was built above another, to the height of seven tiers. Mr. S. adds, that this series of buildings was capable of containing five thousand families, and he gives his reasons for supposing it to be, not a granary of the Incas, as some travellers have imagined, but the residence of the Lord of Chicama, when he resided in the interior of his territory, before it became subject to the Inca Pachacutec. These ruins, it seems, present no remains of delicate sculpture, although some of the stones are carved in arabesques. The remains of the fortified palace of Paramonga, are said to be similar to those of the Lord of Chicama's baronial residence! We half envy Mr. S. of the pleasure he would experience when supposing the antiquity of this Cyclopian structure, and we felicitate him on the delight he must have enjoyed on perfecting his supposition that an edifice, so grand in design and architecture, was the domicile of a sovereign, and not a granary.

Thus have we furnished a distinct and complete exhibition of Dr. Morton's inquiry, under all its discursiveness; and, having weighed his facts and reasons with attention and impartiality, we unreservedly coincide with him in affirming these results:—that, at certain specified places in Peru, there are sculptural and architectural remains: that, in a certain desert, there is a Peruvian cemetry containing numerous sepulchral relics of a people whose badly-formed skulls are small, greatly elongated and narrow in their whole length, with a very retreating forehead: that, in the upper provinces of Peru, there are " innumerable tombs" containing the mortal remains of men, the formation of whose crania seems to prove that they are an extinct race of natives: that, in those tombs, a traveller found skulls remarkable for their extreme extent behind the occipital foramen, with the facial bones much elongated, so that the general appearance of their possessors must have been rather that of the ape-family than of human beings: that, although living in the utmost barbarism at the advent of the Incas, yet the Yuncas had erected a remarkable temple: that the Chimuyans appear to have possessed extensive and regular edifices, with some other attributes of civilization: and that, among the tribes whom the Incas found in occupation of the Peruvian territories, the inhabitants of Collao and Chimù might have been as brave as bull-dogs and as unyielding as the Hurons, without evincing an ordinary degree of intelligence. At the same time, we do not see that the doctor has ventured to determine, or succeeded in adducing evidence to prove, that the people deposited in graves of sand and salt at Atacama, or the ape-like race entombed at Titicaca, could have been endowed by nature with powers capable of designing and erecting the fore-described structural and architectural remains: far less even, has he said or shown that such heads ever devised or directed the construction of the works whose ruins are so interestingly depicted on his pages, as to fascinate the attention of artists and antiquaries. On the belief that the typal skull is quite natural and free from artificial distortion, we conclude, that such a people never did or could produce such works: that the excavations, structures, monuments and sculptures were the workmanship of a race anterior to that which preceded the Incas: and that the men, whose corpses now moulder in the tombs of Titicaca, profaned the fabric of a nobler race by appropriating them for sepulchres.

We abstain purposely from discussing the professor's inquiry as a dialectical or archæological theme, solely because we feel that such would be a misplaced or unfit subject for the Medico-chirurgical Journal. We may just observe however, that he might have described the form of head given to the various human figures and the stone-idols, the fashion of their long vestments, and the kind of architecture adopted in the gateways and porches, the gigantic pyramid and grand monuments resembling Cyclopian remains. But passing this defect of information, we undertake to show physiologically, that such people, *naturally* having small narrow heads, with two-thirds of the brain deposited behind the occipital foramen, are utterly incapable of producing works not unworthy of the arts of ancient Greece or Rome, for grandeur of design and architecture.

Physiologists universally consider the brain as the organ of the mind; and, on broad intelligible grounds, the most of them hold the beautiful and consistent doctrine—that particular distinguishable parts of the brain are the organs of particular distinct faculties of the mind: daily "experience proves that the mind manifests a plurality of faculties by a plurality of organs." Moreover, it is a fundamental physiological doctrine—that, while every organ performs exclusively one proper function, the power of every function, vital or mental, is naturally and principally commensurate with the size of its organ: in other words, that organic size contributes chiefly to functional energy. Hence, this rule being certain, it is most probable that the small skulls, with their tiny brains, in the ancient Peruvian heads, could never have perceived beauty in fine statuary or grandeur in exquisite architecture. There is this other physiological doctrine generally admitted, that the frontal portion of the brain is the seat of the intellect —composes the higher organs of the intellectual faculties. Hence again, it is most probable that the low, narrow, rapidly retreating forehead in this people never could conceive any work in architecture or sculpture, grand in design and magnificent in execution.

This principle of the physiologists seems to pervade universal nature, as an elementary law; and, as it has an intimate connexion with the subject of this article, we would introduce some brief notes in evidence of its applications to the Human Head, and the effects of its size on the degrees of mental vigour.

*First.*—When the head is below a certain size, the person always proves imbecile or idiotic; and this fact holds good in all the existing races of mankind. Thus, for instance, in the lowest class of idiots, where the intellectual manifestations are null, the horizontal circumference, taken a little higher than the orbits, varies from eleven to thirteen inches; while the distance from the root of the nose backwards, over the top of the head to the occipital spine is only between eight and nine inches: this fact is absolute and will bear the test of actual experiment. If therefore, extreme defect of size in the head or brain be invariably accompanied by mental imbecility, it is fair to infer that size will influence the power of mental manifestation, through all other gradations of magnitude—always assuming other conditions to be equal.

*Second.*—As we have seen in Dr. Morton's introductory essay, the ancient Egyptians formed a remarkable variety of the Nilotic family, having the forehead narrower and less elevated than it is in the proper Caucasian head: now, the fact is worthy of attention, that the same dimensions of the anterior cerebral lobe that prevailed in this primitive people, continue to be discernible in nations who, like them, construct great works of art.

*Third.*—The busts and statues of the Greeks and Romans show, that eminent intellectual power was concomitant in them with large anterior cerebral lobes, as a national character.

*Fourth.*—The works of illustrious Painters evince their conviction of the existence of this natural law. For, in their composition-pieces, they constantly exhibit heads possessing a splendid moral and intellectual endowment conjoined with a magnificent development of height and breadth in the middle and anterior cerebral lobes. It is thus that the head of Jesus the All-perfect One is figured in the best pictorial representations of the Redeemer. Had the artists painted Him with a head like the Ancient Peruvians, every European, every enlightened mind, would have turned from the pictures with disgust.

*Fifth.*—It is matter of direct observation and fact—that, in the existing races, European and American, the large anterior cerebral lobe is always co-existent with powerful intellectuality, and smallness of the same lobe is as constantly associated with intellectual imbecility.

*Sixth.*—From these and other facts, we derive authority for this induction, as the principle of a natural law—that the *Size* of all organs determines the first measure of their *Power* in action: but, when practically applying this principle in the Comparison of Size, the scholastic rule—*Cæteris Paribus*, ALL OTHER CONDITIONS BEING EQUAL—must never be overlooked; for, otherwise, the comparison will be inaccurate, and may prove the cause of pain, the support of injustice or the source of error. Again, as every upright and considerate observer must discern, the influences of organic size in affecting the degrees of functional power are manifestly susceptible of modification—by age, health, temperament or constitution, excitement, exercise and education, which increase the aptitude and activity of functional action. Further and finally, according to this Law of Nature, the ancient Peruvians with their heads natural, as figured by Dr. Morton in his typal skull, could not have been, by any possibility, the constructors of the works impliedly ascribed to them; but, if they really did design and rear these works, and had heads depressed and denaturalized by mechanical appliances—as we can discover much reason to suspect it was—then had this people their heads converted into monstrosities, and consequently perverted into objects not amenable to this or any other law for directing the judgment of Nature.

From Dr. Morton's sketches of the Inca-Peruvians, we are furnished with a perspicuous, though compendious, view of their traditionary origin, their establishment in the territories of their predecessors, their warlike and peaceful achievements, their mode of moulding the head into artificial forms, their funeral rites, manners, laws and religion. He figures eight skulls of this nation, chiefly from the cemetery at Pachacamac, or temple of the sun; and at p. 132-3, he gives the result of the measurement of twenty-three adult skulls of the pure Inca race. According to this result, the *largest* of these crania, gives an internal capacity of 89.5 cubic inches, which is a fraction short of the Caucasian *mean*; while the smallest head measures but 60 cubic inches: the *mean* of the whole series gives but 73 cubic inches, which is probably lower than that of any other people now existing. The anterior chamber gives a mean measure of 32 cubic inches—the highest being 36.5, the smallest 23 cubic inches: the posterior chamber gives a mean of 42 cubic inches—the highest being 55.5, the smallest 30 cubic inches: the Coronal region gives 12 cubic inches as a mean—the highest being 20.5, the smallest 9.25 cubic inches: and the mean Facial angle is 75°—the largest being 80°, the smallest 72 degrees. Hence, therefore, the internal capacity of the skull, in the demi-civilized Peruvians, is much less than that of the barbarous nations: it indicates a larger share of intellectuality, by its relative proportions. It is remarkable however, that the doctor found the heads of nine Peruvian children to be as large as those of children of other nations, at the same age; though no specimen among the entire series of thirty-five adult skulls reaches the European average of ninety cubic inches of internal capacity.

Among the forest-solitudes at the sources of the Orinoco, the Baron Humboldt discovered the cavern-sepulchre of the Atures, an extinct but once powerful tribe. Dr. M. describes this remarkable cemetery in the words of its distinguished discoverer: he figures one skull taken from the remains deposited in this cavern; and, according to this representation, the head is more elongated, and less flattened in the occipital region, than is usual in the American race; but the forehead is retreating, the face large, the jaw ponderous and the cheek-bones prominent, as is common to that people. The Aturian is remembered as a numerous and warlike nation, speaking a peculiar language.

Next in order, we come to a cranium of the Puelchés a Patagonian tribe; and, in the plate, we recognize the indications of a formidable savage, in the fulness of development above the opening of the ears, the low forehead, the flattened occiput, the arching of the zygoma, the projecting upper jaw and the broad face. The Puelchés are proverbially brave and skillful in war, as their protracted bloody contests with the Spaniards bear testimony.

On the figured skull of a Charrua, we observe a very broad head, with its retreating forehead and flattened occipital region—a cranium exhibiting the characteristics of the American Indian in very strong relief. The Charruas of Brazil hold the head erect, with a bold physiognomy and fierce countenance, indicative of their haughtiness, ferocity and indomitable spirit: they are cruel, revengeful and exterminating in their wars: all their energies, mental and physical, are devoted to martial pursuits: they have no religion, no laws, no rewards, no punishments, no gradations of rank. Equally horrid is the large ponderous skull of a Brazilian Botocudo approaching, in general aspect, much nearer to that of the Orang-Outang than any other skull from a barbarous nation. From this figure, the mental characters of the breed may be readily ascertained: but, on the Professor's page, they do not appear, for want of information concerning the habits of this people, and their history.

We have an excellent Sketch of the Mexican history and characteristics, p. 141 152, illustrated by eight engravings of skulls, and four wood-cuts representing heads enormously elevated by means of artificial compression from back to front, with high broad foreheads, oval faces, prominent cheek-bones, and tumid lips. The Professor considers the skull figured on his sixteenth plate as a relic of the genuine Toltecan stock: it was exhumed from an ancient cemetery near Mexico; and, from its having been accompanied by numerous antique vessels, weapons, and other remains, he assigns it to a person of distinction. This cranium has large and massive developments; with a full, broad but retreating forehead, and great width between the parietal bones. The head is more oval and elongated than is common in this race, and there is a remarkable fulness in the phrenological region of constructiveness. In accordance with these indications, the Mexicans are known to have excelled the neighbouring nations in architecture, astronomy, arithmetic and metallurgy. They had an established religion, but it was superstitious, idolatrous, and sanguinary: they practised agriculture, and were versant both with the useful and ornamental arts: they had literature, hieroglyphics and a history.

Dr. Morton believes that the Natchez were a branch of the great Toltecan family. They worshiped the sun by keeping an eternal fire; they practised human sacrifices on the death of eminent persons ; and they had hereditary distinctions and fixed institutions. They were more pacific than most of the American tribes: they rarely made wars: they placed not their glory in destroying their fellow-creatures : but when once excited to revenge by repeated provocations, their resentment was appeased only by the extermination of their enemies. About a century ago, they were overpowered and massacred or enslaved by the French; and the Natchez as a nation became extinct. This people was addicted to the practice of human sacrifices to a most frightful extent : sometimes more than a hundred victims were immolated at the funeral of a Great Sun or sovereign chief. Among other singular customs of the Natchez, was that of distorting the head by compression so as to raise it to an incredible height: the process is described by Dr. Morton, and he exhibits two figures—a profile and front view of one of these most singular monstrosities.

One skull of the Chetimaches is figured by Dr. M. and it represents a remarkably massive development: the nearly vertical occiput, the great height of the cranium, and the size and strength of the bones of the face, are not surpassed, he says, by any Indian skull he had ever seen. This nation was powerful and warlike but not numerous : it has disappeared for the last hundred years.

There is an historical sketch of the Seminoles or wanderers, at p. 164 of Dr. M's. descriptions, and he figures four skulls in illustration of the national head. This is said to be a remarkably tall, well-shaped, and very hardy race; the men are affectionate to their wives and children, hospitable to strangers, and not deficient in justice, gratitude and understanding. Nevertheless, they are characterized as proud and arrogant, valiant in battle ; ambitious of conquest, restless and perpetually exercising their arms ; yet magnanimous and merciful to a vanquished enemy, when he submits, and seeks their protection and friendship. In the Seminoles, the head is large, with a lofty though retreating forehead, great breadth between the parietal bones, and remarkable altitude of the whole cranium. The average internal capacity of the Seminole skulls is unusually large, being 87.5 cubic inches, which is a near approach to that of the Caucasian race.

The Cherokees are a very tall, large, robust race of men ; grave and circumspect in their deportment; slow and reserved in their conversation. The arts of peace are more congenial to their disposition than those of war: they are docile, intelligent and capable of instruction, they possess a written language, and many of them have become agriculturists. Dr. M's. plate represents the skull of a Cherokee warrior ; but there is nothing particular in its conformation : its internal capacity is 82. cubic inches.

We may group the skulls figured on Plates XXVII—XXXIV : they exhibit the crania of an Uchee, a Chippeway, a Menominee, a Miami, an Ottigamie, a Potowatomie, a Naumkeag, and a Lenapé or Delaware—tribes in whom the true Indian head prevails, and the Indian disposition is perfectly displayed. Another group, and the next in order, is that of the Iroquois or Mingoes, possessing the strongest traits of skull and character for which an American savage has ever been distinguished. It comprises crania of the Cayuga, Oneida, Huron, Pawnee, Dacota or Sioux, Osage and Cotonay or Blackfoot tribes.

There are several nations of Indians located on the Columbia river, and they have been denominated the Flat-Heads, from their abominable practice of producing, by mechanical contrivances, a depression of the forehead and the consequent elongation of the whole head, until the top of the cranium becomes a nearly horizontal plane, in extreme cases. We have a good description and figure, at Dr. M's. p. 203-4, by which this strange process is accomplished. This family of Flat-Heads consists of the Chinouks, Klastonis, Killemooks, Clatsaks, Kalapooyahs, Clickitats and Cowalitsks: with good figures of the others : two of the Chinouk and two of the Cowalitsk skulls are exhibited by

Dr. M. in all their grotesque and frightful deformity: by this we are told, the absolute internal capacity of the skull is not diminished, and the intellectual faculties suffer nothing: and why should they? Their organs are not lessened; they are merely pushed into an unnatural shape by displacement. Altogether, the head is large in this group, and the skull capacious. These Flat-Heads are inquisitive and loquacious; not deficient in acuteness of understanding; they have retentive memories; and, though fond of feasts and generally cheerful, they are never gay: they are cunning and cautious, mild and inoffensive, and disposed to friendship; in traffic, they are keen and intelligent, and employ much dexterity in chaffering.

Under a section headed "Skulls from the Tumuli or mounds," Dr. Morton takes occasion to inquire into the geographical distribution of these mounds, their uses, and the race of people by whom they were constructed. His chief step in this inquiry consists in an examination of a series of thirteen skulls, obtained in localities remote from each other, but perfectly authenticated by the places and circumstances in which they were procured. This is the list—one from Circleville, in *Ohio;* one from the upper Mississipi; one from the Grave Creek, *Virginia;* one from the Alabama river; one from Tenessee; one from Santa, in *Peru;* two from the valley of Rimac, in *Peru;* three from Otumba, in *Mexico;* one from Golconda, in *Illinois;* and one from Steubenville, in *Ohio.* Of this series, the first twelve have the low forehead, high cheek-bones, small facial angle, massive lower jaw, prominent vertex, flat occiput, and rounded head of the American Race: with reference, therefore, to these physical characters and to the geographical distribution of the skulls, Dr. M. considers them as remains of the Toltecan family, and he thinks it probable that all the tribes which erected mounds as a national usage, were branches of the great Toltecan stock. The cranium from Steubenville is set down as having certainly belonged to an individual of the Muskogees, or other barbarous tribe.

Two representations of the skulls of Charibs, from Venezuela and St. Vincent, occupy the professor's sixty-fourth and sixty-fifth plates. In this people, the head is naturally rounded, but many of the Charib nations practise the flattening process, in such a manner as to depress the frontal bone, and thus to elongate the skull from front to back: the disfigurement thus produced is manifest in the first of these drawings; in the second, it is excessive. This miserable custom becomes yearly less prevalent; and thus, by opportunities not distant, the physiologist will be enabled to contemplate the Charibbean head under its natural configuration. The Charibs were the most ferocious and brutal of the American nations: they were without laws and almost devoid of religious observances: they conducted all their enterprizes with extraordinary craftiness: they were suspicious and revengeful, morose and melancholy: their cannibalism was execrable: they looked upon all other men as mere beasts fit only to be slain and devoured.

On the plates LXVI. LXVII. and LXVIII. we find three figures of the Arancanian skulls: the two first exhibit a side and front view of the cranium of Bampuni, a chief who was slain in battle, in 1835; the last is from the head of Chilicoi, another chief who fell in the same encounter. Bampuni's skull is symmetrical, with the frontal bone lofty but narrow: the whole posterior cranium is full, and the internal capacity not much short of the Caucasian mean. We are struck with the projecting face of Chilicoi and the consequent small facial angle; with the lowness of his forehead, his flattened vertex, and the smallness of his whole head. Dr. Morton's collection contains three Arancanian skulls, and he finds their mean internal capacity to be 79 cubic inches, with an average facial angle barely seventy-five degrees. Hence, the national head is larger than the Peruvian, and somewhat less than the mean of the collective American race. The Arancanians are a robust and muscular people, endowed with extraordinary bodily activity, brave, discreet, cunning to a proverb, patient in fatigue, enthusiastic in all their undertakings, chivalrous and fond of war as the only source of distinction. They had invented numbers, and possessed the useful arts before their intercourse with Europeans: they are highly susceptible of mental culture, but despise the restraints of civilization; and those of them, who have been educated in the Spanish colonies, embraced the first opportunity to resume the habits of their nation.

As an additional evidence of the unity of race and species in the American nations, Dr. Morton adduces the singular fact—that, from Patagonia to Canada, from ocean to ocean, in the civilized and uncivilized tribes, a peculiar mode of placing the body in sepulture, has been practised from immemorial time. This peculiarity consists in depositing the corpse in a *sitting posture*, and this is represented most graphically on his sixty-ninth plate, in the figure of a natural mummy. He is aware that this custom is not exclusively American; but the evidences and arguments which support his proposition are abundantly conclusive.

Four genuine skulls of the Esquimaux, or Mongol-Americans, are engraved on the seventieth plate; and, while they show the great and uniform differences between the heads of this people and those of the American Indians, they also serve as a physical (and a mental, as we would add) corroboration of the opinion —that the Esquimaux are the only people possessing Asiatic characteristics on the American continent.

As an Appendix to his Cranioscopy, the doctor introduces an Essay intitled "Phrenological Remarks on the relation between the natural talents and dispositions of nations and the development of their brains." It was contributed, as Dr. M.'s request, by Mr. Combe; and, like all this writer's compositions, it is distinguished by elegance, conciseness, and perspicuity, by depth of thought, soundness of judgment, generosity of feeling, and acuteness of ratiocination. Dr. Morton has illustrated this sketch with the engraving of a European skull, which is outlined into the three regions of cerebral organs—the animal, moral and intellectual—corresponding and subservient to the three orders of mental faculties. He has also illustrated the "Phrenological Remarks" with a most elaborate and comprehensive "Table of the Phrenological Measurements" of one hundred skulls, showing the dimensions of each head and of the several cerebral organs assigned to co-existing faculties of the mind: but, important as this table is, it would have been greatly more valuable if so many of its subjects had not been denaturalized by the barbarous fashion of disfiguring the head with artificial appliances.

Here, we finish our account of Dr. Morton's American Cranioscopy; and by its extent and copiousness, our article will show how highly we have appreciated his classical production: we have studied his views with attention, and examined his doctrines with fairness; and, with perfect sincerity in rising from a task which has afforded unusual gratification, we rejoice in ranking his "CRANIA AMERICANA" in the highest class of transatlantic literature, forseeing distinctly that the Book will ensure for its author the well-earned meed of a *Caucasian* reputation.

